# Management of Type 2 Diabetic Kidney Disease in 2022: A Narrative Review for Specialists and Primary Care

**DOI:** 10.1177/20543581221150556

**Published:** 2023-01-25

**Authors:** David Z. I. Cherney, Alan Bell, Louis Girard, Philip McFarlane, Louise Moist, Sharon J. Nessim, Steven Soroka, Sara Stafford, Andrew Steele, Navdeep Tangri, Jordan Weinstein

**Affiliations:** 1 Division of Nephrology, Department of Medicine, Toronto General Hospital, University of Toronto, ON, Canada; 2 Temerty Faculty of Medicine, University of Toronto, ON, Canada; 3 Department of Family & Community Medicine, University of Toronto, ON, Canada; 4 Division of Nephrology, Department of Medicine, Cumming School of Medicine, University of Calgary, AB, Canada; 5 Division of Nephrology, Department of Medicine, Schulich School of Medicine & Dentistry, Western University, London, ON, Canada; 6 Division of Nephrology, Jewish General Hospital, McGill University, Montreal, QC, Canada; 7 QEII Health Sciences Centre, Nova Scotia Health, Halifax, Canada; 8 Fraser Health Division of Endocrinology, University of British Columbia, Surrey, Canada; 9 Lakeridge Health, Whitby, ON, Canada; 10 Departments of Medicine and Community Health Sciences, University of Manitoba, Winnipeg, Canada; 11 Division of Nephrology, St. Michael’s Hospital, University of Toronto, ON, Canada

**Keywords:** Canadian, chronic kidney disease, type 2 diabetes, SGLT2i, finerenone

## Abstract

**Purpose of review:**

Kidney disease is present in almost half of Canadian patients with type 2 diabetes (T2D), and it is also the most common first cardiorenal manifestation of T2D. Despite clear guidelines for testing, opportunities are being missed to identify kidney diseases, and many Canadians are therefore not receiving the best available treatments. This has become even more important given recent clinical trials demonstrating improvements in both kidney and cardiovascular (CV) endpoints with sodium-glucose cotransporter 2 (SGLT2) inhibitors and a nonsteroidal mineralocorticoid receptor antagonist, finerenone. The goal of this document is to provide a narrative review of the current evidence for the treatment of diabetic kidney disease (DKD) that supports this new standard of care and to provide practice points.

**Sources of information:**

An expert panel of Canadian clinicians was assembled, including 9 nephrologists, an endocrinologist, and a primary care practitioner. The information the authors used for this review consisted of published clinical trials and guidelines, selected by the authors based on their assessment of their relevance to the questions being answered.

**Methods:**

Panelists met virtually to discuss potential questions to be answered in the review and agreed on 10 key questions. Two panel members volunteered as co-leads to write the summaries and practice points for each of the identified questions. Summaries and practice points were distributed to the entire author list by email. Through 2 rounds of online voting, a second virtual meeting, and subsequent email correspondence, the authors reached consensus on the contents of the review, including all the practice points.

**Key findings:**

It is critical that DKD be identified as early as possible in the course of the disease to optimally prevent disease progression and associated complications. Patients with diabetes should be routinely screened for DKD with assessments of both urinary albumin and kidney function. Treatment decisions should be individualized based on the risks and benefits, patients’ needs and preferences, medication access and cost, and the degree of glucose lowering needed. Patients with DKD should be treated to achieve targets for A1C and blood pressure. Renin-angiotensin-aldosterone system blockade and treatment with SGLT2 inhibitors are also key components of the standard of care to reduce the risk of kidney and CV events for these patients. Finerenone should also be considered to further reduce the risk of CV events and chronic kidney disease progression. Education of patients with diabetes prescribed SGLT2 inhibitors and/or finerenone is an important component of treatment.

**Limitations:**

No formal guideline process was used. The practice points are not graded and are not intended to be viewed as having the weight of a clinical practice guideline or formal consensus statement. However, most practice points are well aligned with current clinical practice guidelines.

## Abrégé

**Justification:**

L’insuffisance rénale est présente chez près de la moitié des patients canadiens atteints de diabète de type 2 (DT2). Il s’agit également de la première manifestation cardiorénale la plus fréquente du DT2. Bien qu’il existe des lignes directrices claires pour son dépistage, des occasions de diagnostiquer l’insuffisance rénale sont manquées, ce qui fait en sorte que de nombreux Canadiens ne reçoivent pas les meilleurs traitements disponibles. Cette préoccupation a pris de l’importance puisque de récents essais cliniques ont démontré des améliorations dans les paramètres rénaux et cardiovasculaires (CV) avec la prise de finérénone, un antagoniste non stéroïdien des récepteurs minéralocorticoïdes (nsMRA), et d’inhibiteurs du cotransporteur de glucose de sodium 2 (SGLT2). L’objectif de cet article est de fournir une revue narrative des données probantes actuelles appuyant cette nouvelle norme de soins pour le traitement de l’insuffisance rénale diabétique (IRD), ainsi que des points de pratique.

**Sources de l’information:**

Un groupe d’experts composé de cliniciens canadiens, dont neuf néphrologues, un endocrinologue et un prestataire de soins primaires, a été formé. Les auteurs de cette revue ont utilisé des lignes directrices et des essais cliniques publiés comme sources; ceux-ci ont été choisis sur la base d’une évaluation de leur pertinence pour les questions auxquelles ils avaient répondu.

**Méthodologie:**

Les panélistes se sont réunis virtuellement pour discuter de potentielles questions à répondre dans le cadre de cette revue, et se sont entendus sur dix questions clés. Deux membres du panel se sont portés volontaires pour être co-responsables et rédiger les résumés et les points de pratique pour chacune des questions identifiées. Ces derniers ont été distribués par courriel à l’ensemble des auteurs. Après deux tours de vote en ligne, une deuxième réunion virtuelle et la correspondance électronique qui a suivi, les auteurs sont parvenus à un consensus sur le contenu de la revue narrative, y compris sur tous les points de pratique.

**Principaux résultats:**

Il est essentiel que l’IRD soit diagnostiquée le plus tôt possible afin de prévenir de façon optimale la progression de la maladie et les complications qui y sont associées. On devrait procéder au dépistage systématique de l’IRD chez les patients diabétiques par l’évaluation de l’albumine urinaire ET de la fonction rénale. Les décisions relatives au traitement devraient être individualisées en fonction des risques et des avantages pour le patient, de ses besoins et préférences, de l’accès aux médicaments et des coûts, ainsi que du degré nécessaire de réduction de la glycémie. Les patients atteints d’IRD devraient être traités pour atteindre les cibles d’A1c et de pression artérielle. Le blocage du SRAA et le traitement avec des inhibiteurs du SGLT2 sont également des composantes clés de la norme de soins visant à réduire le risque d’événements rénaux et CV pour ces patients. La finérénone devrait également être envisagée pour réduire encore davantage les risques d’événements CV et de progression vers l’IRC. L’éducation des patients diabétiques auxquels on prescrit des inhibiteurs du SGLT2 et/ou de la finérénone est un élément important du traitement.

**Limites:**

Aucun processus officiel de directives n’a été utilisé. Les points de pratique ne sont pas notés et ne sont pas destinés à être considérés comme ayant le poids d’une directive de pratique clinique ou d’une déclaration de consensus officielle. Cependant, la plupart des points de pratique sont bien alignés avec les lignes directrices actuelles de pratique clinique.

## Introduction

Kidney disease is a relatively common complication of type 2 diabetes (T2D), present in almost half of Canadian patients with T2D.^1^ Almost half of new dialysis cases in Canada are patients with diabetes.^2^ Kidney disease is also the most common first cardiorenal manifestation of T2D.^3^ Despite clear guidelines for testing, many opportunities are being missed to identify kidney disease in people with diabetes, and many Canadians are therefore not receiving optimal management of their diabetes.^4,5^ This has become more important given recent clinical trials demonstrating improvements in both kidney and cardiovascular (CV) outcomes with sodium-glucose cotransporter 2 (SGLT2) inhibitors and the nonsteroidal mineralocorticoid receptor antagonist (nsMRA) finerenone in the context of T2D. These trials signal a new standard of care in diabetic kidney disease (DKD).^6-8^

The goal of this document is to provide a narrative review of the evidence supporting a new standard of care within the Canadian treatment landscape.

## Methods

An expert panel of Canadian clinicians was assembled, including 9 nephrologists, an endocrinologist, and a primary care practitioner. The panelists were identified and invited by Dr. David Cherney, based on their expertise and interest in this subject area. This panel met in 2021 and 2022 to develop a series of evidence-informed practice points for the optimal management of DKD, with the goal of improving care by Canadian physicians and optimizing outcomes for their patients. The entirety of the review was developed by this panel, with no input from any other outside parties.

At a meeting on October 5, 2021, the panel presented, debated, and reached consensus on 10 key questions considered to be clinically relevant to integrating best evidence to the new standard of care for DKD. Two panel members were co-leads for each of the identified questions. Based on the available data, guidelines, and their own experience, the leads developed summaries of the pertinent information and proposed 1 or more practice points pertaining to the relevant question. Completed summaries and practice points were shared with the entire panel, and individual members voted on whether they agreed or disagreed with the wording of the practice points. The panel members discussed the manuscript during a second meeting held on April 1, 2022, and the updated practice points went to a second vote. Content that did not receive unanimous consensus among the authors was further reviewed and revised until consensus was reached. The updated manuscript was circulated to the entire committee for a final vote, and all authors agreed with the content, including all the practice points.

## Review

### Question 1. What Is the Importance of Early Identification of DKD and How Are We Performing in Canada?

The identification of DKD is of critical importance to the overall health of people with diabetes.^4,9^ DKD is highly prevalent among people with diabetes; a recent Canadian study showed a DKD prevalence of 47.9% among more than 31 000 people with T2D seen by an endocrinologist.^1^

DKD is usually progressive, with later stages associated with significant kidney-related and CV morbidity and mortality.^4,9^ Across the spectrum of disease severity, there are a number of effective interventions that can attenuate disease progression and prevent cardiorenal complications, with more options available at earlier stages of the disease.^9^ These include lifestyle modifications and smoking cessation, although the evidence for these largely comes from observational studies.^10-14^ Evidence-based standard of care includes renin-angiotensin-aldosterone system (RAAS) inhibition,^15^ optimal blood pressure (BP) control,^16^ optimal blood glucose control,^17^ and statin therapy.^18^

Newer interventions, such as SGLT2 inhibitors and finerenone, which are reviewed in more detail below, offer additional cardiorenal protection when added to RAAS inhibition.^6-9,19,20^ It is critical that DKD be identified as early as possible in the course of the disease to optimally prevent disease progression and associated complications.^4^

**Figure 1.**
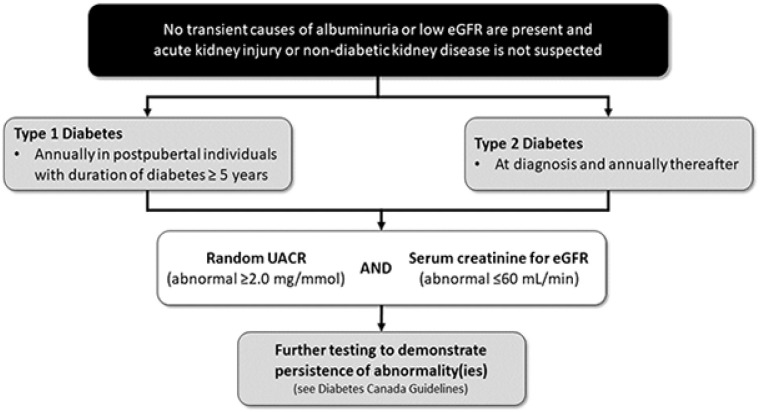
Screening recommendations for early identification of kidney disease in patients with T2D. *Source.* Adapted from Diabetes Canada Clinical Practice Guidelines, 2018.^4^ *Note.* T2D = type 2 diabetes; eGFR = estimated glomerular filtration rate; UACR = urine albumin-to-creatinine ratio.

The recommendations for screening for DKD are described in Canadian and international guidelines.^4,21,22^ The Diabetes Canada clinical practice guidelines recommend that screening for DKD include both an assessment of urinary albumin excretion (typically through a random urine albumin-to-creatinine ratio [UACR]) and a measurement of the kidney function through an estimated glomerular filtration rate (eGFR), using serum creatinine and clinical characteristics (Figure 1).^4^ For people with T2D, screening should be done at diagnosis and at least annually thereafter.^4^ For those with type 1 diabetes, screening is recommended annually for postpubertal individuals with a disease duration of at least 5 years.^4^ Note that initial abnormal tests need to be confirmed to demonstrate persistently elevated UACR and/or persistently low eGFR.^4^ These recommendations have been largely unchanged across clinical practice guidelines in diabetes over the past 2 decades.^4,23-25^

In Canada and worldwide, statistics indicate that uptake of guideline-recommended screening is suboptimal. A retrospective, longitudinal study of 2399 patients with newly diagnosed diabetes from 18 primary-care practices in Southwestern Ontario from 2009 to 2014 assessed the extent to which screening for DKD in these practices matched guideline recommendations. The study showed that 144 (6%) of the cohort had both UACR and eGFR completed within the first year after diagnosis, 170 (7%) were screened with UACR alone, and 1292 (54%) were screened with eGFR alone. A further 793 (24%) had neither screening test completed within the first year of diagnosis.^26^ In a pan-Canadian cross-sectional study using data from the Canadian Primary Care Sentinel Surveillance Network from 2010 to 2015 (N = 46 162), among patients with diabetes who were diagnosed with chronic kidney disease (CKD), only 32.4% had received a UACR test within 6 months of the initial eGFR.^5^ Similarly, an analysis of routine laboratory and administrative data in Alberta from 2015 to 2017 by the Kidney Health Strategic Clinical Network showed that only 42.6% of adults with diabetes had at least 1 documented albumin-to-creatinine ratio test.^27^ These rates were similar in magnitude to the 35% prevalence of albuminuria screening in a multinational study from the CKD Prognosis Consortium,^28^ highlighting that the lack of appropriate DKD screening is a wider problem internationally.

There exists a clear need to enhance screening practices for DKD, recognizing the importance of regular guideline-recommended use of both eGFR and albuminuria testing for all patients with diabetes. The emergence of effective disease-modifying treatment options (ie, SGLT2 inhibitors and finerenone) providing cardiorenal protection for individuals with DKD has made the timely identification and treatment of this common comorbidity even more critical.

**Practice Point:**

Patients with diabetes should be routinely screened for DKD with assessments of urinary albumin and kidney function, following current Diabetes Canada guidelines.

### Question 2. Does the Historic Standard of Care in DKD Remain Relevant in Light of Recent Advances?

While the central theme of this narrative review is to discuss the role of new therapies (ie, SGLT2 inhibitors, finerenone) in the management of DKD, it is critical to reinforce the continued importance of the historic standard of care (glycemic control, BP control, and RAAS blockade). The new therapies have demonstrated CV and kidney protection *in addition to* the established standards of care. This section provides a review of the guideline recommendations and rationale for the historic standard-of-care therapies in DKD and their place in the recent landmark trials supporting the use of SGLT2 inhibitors and finerenone.

#### Glycemic control

The Diabetes Canada clinical practice guidelines recommend that all people with diabetes be treated to achieve optimal control of blood glucose to prevent the onset and delay the progression of CKD.^4^ The target recommended in the guidelines is lower than 7.0% for most people, with lower or higher targets being appropriate based on individual patient characteristics. The glucose targets that have been established are largely based on findings from large clinical trials, including the landmark UKPDS study showing better kidney outcomes among patients with more intensive glucose control.^29^ In fact, published longer term follow-up from the ADVANCE trial demonstrated a reduction in progression to end-stage kidney disease (ESKD).^30^ The mechanisms of benefit for glycemic control are thought to include a reduction in the production of advanced glycation end products and activation of their receptor, with reduced downstream generation of reactive oxygen species and a reduction in inflammation.^31^ It is important to note that intensive glycemic control was associated with harm in these trials, which appeared to be driven predominantly by hypoglycemic events. These findings may have limited the full uptake of intensive glucose control, but these trials were also limited by the agents that were available. The newer antihyperglycemic agents (SGLT2 inhibitors, glucagon-like peptide-1 receptor agonists [GLP1-RAs], and dipeptidyl peptidase-4 inhibitors [DPP4is]) typically have very low rates of hypoglycemia, and, therefore, intensive glucose control may be more safely achieved.^32^ The ranges of expected A1C reduction for SGLT2is and DPP4is are in the range of 0.5% to 0.7%; while for the GLP1-RAs, one can expect A1C reduction in the range of 0.6% to 1.4%.^32^

#### Blood pressure control

The Diabetes Canada guidelines recommend a target BP of <130/<80 mm Hg for most people with diabetes.^33^ Some expert consensus groups have recommended even lower targets. For example, KDIGO’s (Kidney Disease Improving Global Outcomes) 2021 Clinical Practice Guideline for the Management of BP in CKD recommends a target of <120 mm Hg systolic BP for people with hypertension and CKD, with or without diabetes.^34^ While guidelines acknowledge that demonstration of kidney protection with BP control has not been consistent across clinical trials,^4^ multiple studies (eg, ABCD, ACCORD BP) have demonstrated a reduction in the development of microalbuminuria or macroalbuminuria with more intensive BP lowering among people with diabetes.^16^ Additionally, post hoc analysis of both the RENAAL and IDNT studies among patients with DKD showed that lower BP was associated with significant reductions in risk of hard kidney outcomes (eg, composite of doubling of serum creatinine, ESKD, or death).^35,36^

#### RAAS inhibition

The use of an angiotensin II receptor blocker (ARB) or angiotensin-converting enzyme inhibitor (ACEi) is recommended to slow the progression of DKD.^4^ In the literature, RAAS blockade is consistently associated with the attenuation of risk of hard kidney outcomes, such as progression to ESKD or doubling of serum creatinine (eg, the primary outcome findings from RENAAL and IDNT).^37,38^ Kidney protection has been demonstrated in both hypertensive and normotensive albuminuric patients with T2D.^15^ Suppression of the RAAS is believed to directly influence DKD pathophysiology, as both angiotensin II and aldosterone have been directly linked to DKD pathogenesis through a number of different local and systemic mechanisms.^39^

Mineralocorticoid receptor agonists (MRAs), until recently, had not been studied in major cardiorenal outcome trials among individuals with DKD. Although there is some evidence of kidney benefit with the older MRAs like eplerenone or spironolactone (reduction of albuminuria when combined with an ACEi or ARB compared to ACEi or ARB alone), clinical practice guidelines have not recommended these agents as part of the standard of care.^21,40,41^ In contrast, the nsMRA finerenone has been included in the current KDIGO and American Diabetes Association (ADA) guidelines.^21,41^

#### Standard-of-care therapies in trials with SGLT2 inhibitors and finerenone

In the pivotal studies with SGLT2 inhibitors and finerenone that will be examined in greater detail in subsequent sections of this review, the standard-of-care therapies described above were used by the majority of participants at baseline (Table 1).^6,8,19,42,43^ For example, RAAS inhibitors were used in almost all participants across the studies.

**Table 1.** Proportions of Standard-of-Care Therapies at Baseline in Pivotal Trials With SGLT2 Inhibitors and Finerenone in DKD.^6,8,19,42,43^

Class/agent	SGLT2 inhibitors			Finerenone	
Study	CREDENCE	DAPA-CKD (T2D subgroup)	EMPA-KIDNEY (T2D subgroup)	FIGARO-DKD	FIDELIO-DKD
Study treatments	Canagliflozin vs placebo	Dapagliflozin vs placebo	Empagliflozin vs placebo	Finerenone vs placebo	Finerenone vs placebo
Baseline antihyperglycemic use					
Metformin	58%	43%	22%	69%	44%
Sulfonylurea	29%	27%	19%	28%	23%
DPP4i	17%	26%	26%	24%	27%
GLP1-RA	4%	4%	10%	8%	7%
SGLT2i	—	—	—	8%	5%
Insulin	66%	55%	55%	54%	64%
Baseline A1C	8.3%	7.8%		7.7%	7.7%
Baseline RAASi use, %					
ACEi or ARB	99.9%	NR	85%	>99%	NR
ACEi	NR	31%	NR	43%	34%
ARB	NR	67%	NR	57%	66%
Baseline non-RAASi antihypertensive use, %					
Diuretic	47%	50%	54%	48%	57%
β-blocker	40%	NR	52%	48%	52%
CCB	NR	NR	NR	51%	63%
α-blocker	NR	NR	NR	19%	25%
Mean baseline SBP	140 mm Hg	137 mm Hg	139 mm Hg	136 mm Hg	138 mm Hg
Mean baseline DBP	78 mm Hg	77 mm Hg	76 mm Hg	77 mm Hg	76 mm Hg

*Note.* ACEi = angiotensin-converting enzyme inhibitor; A1C = glycated hemoglobin; ARB = angiotensin II receptor blocker; CCB = calcium channel blocker; DBP = diastolic blood pressure; DKD = diabetic kidney disease; DPP4i = dipeptidyl peptidase-4 inhibitor; GLP1-RA = glucagon-like peptide-1 receptor agonist; NR = not reported; RAASi = renin-angiotensin-aldosterone system inhibitor; SBP = systolic blood pressure; SGLT2i = sodium-glucose cotransporter-2 inhibitor; T2D = type 2 diabetes.

Standard-of-care therapies continue to be important pillars of overall cardiorenal risk reduction among patients with DKD. The important benefits associated with the new agents were largely seen when added to standard-of-care therapies.

**Practice Points:**

Patients with DKD should be treated to achieve targets for A1C, following current guidelines from Diabetes Canada.
Patients with DKD should be treated to achieve a target BP following current guidelines from Diabetes Canada and Hypertension Canada.
Patients with DKD should be treated with a RAAS inhibitor (either an ARB or ACEi).

### Question 3. What Is the Evidence Supporting SGLT2 Inhibitors as a New Standard of Care in DKD?

Early indications of potential SGLT2 inhibitor benefits on kidney function came from CV safety trials undertaken in patients with T2D (ie, with canagliflozin, dapagliflozin, and empagliflozin in the CANVAS, DECLARE-TIMI 58, and EMPA-REG OUTCOMES trials, respectively). While the patients enrolled in these trials had CV risk factors, kidney function was largely intact as demonstrated by the relatively high mean eGFR values and low median levels of albuminuria. Even in these patients at relatively low risk of kidney events, exploratory analyses suggested treatment with SGLT2 inhibitors was associated with decreased albuminuria, slowed eGFR decline, and reductions in kidney outcomes such as doubling of serum creatinine (or decrease in eGFR of ≥40%), incident ESKD, and death from renal causes.^44-46^

**Figure 2.**
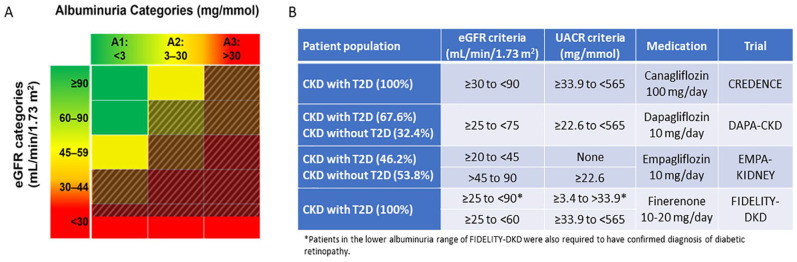
(A) Shaded areas indicate coverage of key kidney measures by inclusion criteria of completed primary kidney outcome trials of SGLT2 inhibitors and finerenone. (B) Table depicting full eGFR and albuminuria inclusion criteria of primary kidney outcome trials in patients with CKD and T2D. *Note.* SGLT2 = sodium glucose cotransporter 2; eGFR = estimated glomerular filtration rate; CKD = chronic kidney disease; T2D = type 2 diabetes; UACR = urine albumin-to-creatinine ratio; DKD = diabetic kidney disease.

Subsequently, clinical trials were undertaken with SGLT2 inhibitors specifically among individuals with CKD. These trials had primary composite kidney outcomes consisting of doubling of serum creatinine (or decrease in eGFR of ≥40%), incident ESKD, or renal or CV death.^6,7^ In the CREDENCE trial, canagliflozin 100 mg daily significantly reduced the incidence of the primary outcome in a population with an eGFR ranging from 30 to 90 mL/min/1.73 m^2^ and UACR >34 to 565 mg/mmol, after a median follow-up time period of 2.6 years.^6^ The DAPA-CKD trial was undertaken in a population either with T2D (67% of patients) or without T2D with comparable levels of kidney disease (eGFR 25-75 mL/min/1.73 m^2^ and UACR >22.6-565 mg/mmol) and showed that treatment with 10 mg of dapagliflozin daily over a median period of 2.4 years also reduced a primary composite kidney outcome.^7^ This effect was consistent regardless of T2D status. The recently completed EMPA-KIDNEY trial evaluated patients with kidney disease regardless of T2D status, among those either with eGFR 20 to <45 mL/min/1.73 m^2^ or with an eGFR 45 to <90 mL/min/1.73 m^2^ and UACR ≥22.6 mg/mmol. Empagliflozin 10 mg daily was associated with a significant reduction in the primary composite outcome (Figure 2B).^43^

In the CREDENCE, DAPA-CKD, and EMPA-KIDNEY trials, treatment with SGLT2 inhibitors also reduced incident ESKD and slowed eGFR decline significantly over the chronic phases of treatment.^6,7,47^ Despite the dip in eGFR associated with SGLT2 inhibitor initiation, the mean eGFR in patients taking SGLT2 inhibitors was higher than that in patients taking placebo at the end of each of the studies. The use of SGLT2 inhibitors was discontinued upon initiation of dialysis in the kidney outcome trials, so no potential benefit has yet been demonstrated in patients with ESKD. Of note, almost all patients in CREDENCE and DAPA-CKD trials, and a significant majority of those in the EMPA-KIDNEY trial, were taking maximally tolerated indicated doses of ACEi or ARB medications (Table 1).^6,7,47^

A meta-analysis published at the same time as the EMPA-KIDNEY trial included patient-level data from all the major placebo-controlled trials conducted with SGLT2 inhibitors up to and including EMPA-KIDNEY.^48^ The key findings from the meta-analysis of these 13 trials (total N = 90 413) were that relative to placebo, treatment with an SGLT2 inhibitor reduced the risk of kidney disease progression by 37% (relative risk [RR] 0.63, 95% confidence interval [CI] 0.58-0.69) with similar RRs in patients with and without diabetes. Furthermore, when the data were restricted to 4 CKD-only trials (the 3 trials discussed in this section plus SCORED [sotagliflozin]), the RRs were similar across relevant subgroups (eg, primary kidney diagnosis, presence or absence of diabetes, and baseline eGFR).

These data support 2 critical points in the use of SGLT2 inhibitors in the treatment of DKD. First, the consistency of the effect on kidney outcomes regardless of T2D status or baseline A1C demonstrates that the kidney benefits are independent of glycemic effects, which is important given that glycemic effects of SGLT2 inhibitors are reduced at lower levels of eGFR. Second, the kidney benefit conferred by SGLT2 inhibitors is additive to that provided by the prior standard-of-care treatment including BP control, A1C control, and use of ACEi or ARB medications.

**Practice Points:**

For patients with DKD and eGFR >20 mL/min/1.73 m^2^, treatment with an SGLT2 inhibitor can be initiated as part of the standard of care along with ACEi/ARB use, BP control, and A1C control.
For patients who progress to ESKD requiring dialysis, SGLT2 inhibitors should be discontinued.

### Question 4. What Other Possible Benefits Are Associated With Using SGLT2 Inhibitors in Patients With DKD?

SGLT2 inhibitors are a good option for glycemic control in patients with T2D, with the added benefit of small reductions in body weight and systolic BP.^32^ However, the magnitude of A1C lowering is reduced as eGFR declines, given the reduced ability of the impaired kidney to excrete glucose into urine. The magnitude of weight loss is also reduced as kidney function declines.^49^ Additionally, there are some indications from large cohort studies and post hoc analyses of randomized controlled trials (RCTs) that treatment with SGLT2 inhibitors may reduce the incidence of gout, occurrence of gout flares, and need for gout treatments in patients with T2D.^50,51^ Initiation of treatment with an SGLT2 inhibitor was also associated with a clinically significant reduced risk of incident and recurrent nephrolithiasis in a cohort study.^52^ Treatment with an SGLT2 inhibitor does not increase the risk of hyperkalemia in patients with T2D and may decrease its incidence in patients with T2D and advanced kidney disease, which may help to maintain or augment treatment with other protective medications such as RAAS inhibitors.^53,54^

More importantly, SGLT2 inhibitors have been shown to reduce hospitalizations for heart failure (HF) across broad populations of patients, regardless of T2D or CKD status. The DAPA-HF and DELIVER trials each enrolled patients with HF with or without diabetes. DAPA-HF was conducted among participants with HF and reduced ejection fraction (HFrEF: left ventricular ejection fraction [LVEF] <40%) and eGFR ≥30 mL/min/1.73 m^2^. DELIVER included participants with HF and preserved ejection fraction (HFpEF: ≥40%) and eGFR ≥25 mL/min/1.73 m^2^. In both studies, dapagliflozin 10 mg daily provided a reduction in the primary outcome of CV death, hospitalization for HF, or urgent HF visit. This reduction was consistent regardless of T2D status or eGFR level.^55,56^ The EMPEROR-Reduced and EMPEROR-Preserved studies both evaluated the effect of empagliflozin 10 mg daily in patients with HFrEF and heart HFpEF, respectively, with the same LVEF thresholds as the dapagliflozin studies. Patients were recruited regardless of diabetes status and if their eGFR was ≥20 mL/min/1.73 m^2^, and active treatment in each study reduced the primary composite outcome of CV death or hospitalization for worsening HF.^57,58^ The CHIEF-HF study demonstrated that canagliflozin 100 mg daily significantly reduces patient-reported symptoms of HF regardless of diabetes status. Importantly, this study was conducted entirely remotely—patients were enrolled and monitored with only virtual visits, and no new safety signals were identified.^59^ Meta-analysis of cardiovascular outcome trials (CVOTs) has demonstrated reductions in hospitalization for HF associated with SGLT2 inhibitor treatment in patients with T2D, including those with and without CKD, and in patients with CKD regardless of T2D status.^6,7,60,61^ Reductions in subsequent hospitalizations with SGLT2 inhibitor treatment have also been demonstrated for patients with acute decompensated HF.^62^

In addition to proven benefits in hospitalization for HF, a reduction in the incidence of 3-point major adverse CV events (MACE: composite of nonfatal myocardial infarction, nonfatal stroke, and CV death) was seen in patients with T2D and CKD in the CREDENCE trial. Importantly, renal and CV benefits were consistent regardless of CV disease history or prior CV events.^6,63^ Results from the CANVAS program show that the CV benefit of canagliflozin 100 mg daily in patients with diabetes is likely to extend across the spectrum of renal function; however, a meta-analysis suggests that benefits on 3-point MACE outcomes may be restricted to patients with established atherosclerotic CV disease in the absence of kidney disease.^64-66^ In a secondary analysis from the DAPA-CKD trial, treatment with dapagliflozin 10 mg daily was shown to reduce the incidence of all-cause mortality in patients with CKD regardless of their diabetes status.^7^

Critically, lower levels of eGFR do not seem to mitigate the benefits of SGLT2 inhibitors on the incidence of kidney events, hospitalization for HF, CV outcomes, or all-cause mortality. Their use should be encouraged in eligible patients with diabetes and kidney impairment to reduce these outcomes regardless of the magnitude of glucose lowering. If further glucose reduction is required to meet glycemic targets, other medications may be added in combination with the SGLT2 inhibitor. While hypoglycemia is uncommon with SGLT2 inhibitors alone, their use in combination with drugs that are associated with increased hypoglycemia risk (eg, sulfonylurea or insulin) may result in an increased risk of hypoglycemia.^32^ SGLT2 inhibitors are also not significantly associated with an increased risk of acute kidney injury (AKI), confirming their safety in patients with CKD with and without diabetes.^6,7,48^

**Practice Points:**

In patients with a history of HF, SGLT2 inhibitors should be initiated to reduce the risk of CV death or hospitalization for HF.
In high-risk patients with T2D (ie, those with atherosclerotic cardiovascular disease (ASCVD) and/or CKD), SGLT2 inhibitors should be initiated to reduce the risk of CV events (Table 2).

**Table 2.** Cardiorenal Trials of SGLT2 Inhibitors and nsMRAs Available in Canada: GFR and UACR Ranges, Statistically Significant Endpoints.^6,8,19,42,43,45-47,55-58,64^

eGFR (mL/min/1.73 m^2^)	UACR (mg/mmol)	Trial	Medication	Primary outcome	Significant secondary outcomes
≥30 to <90	≥33.9 to <565	CREDENCE	Canagliflozin 100 mg	Kidney composite	(CV death or HHF), 3-point MACE, HHF, (ESKD or 2× serum creatinine or renal death)
≥30	n/a	CANVAS	Canagliflozin 100 or 300 mg	3-Point MACE	n/a
≥25 to <75	≥22.6 to <565	DAPA-CKD	Dapagliflozin 10 mg	Kidney composite	(≥50% decreased eGFR or new ESKD or renal death), (CV death or HHF), all-cause mortality
≥60	n/a	DECLARE-TIMI 58	Dapagliflozin 10 mg	3-Point MACE^a^	CV death or HHF
≥30	n/a	DAPA-HF	Dapagliflozin 10 mg	Worsened HF or CV death	(CV death or HHF), change in KCCQ score at 8 months
≥25	n/a	DELIVER	Dapagliflozin 10 mg	Worsened HF or CV death	(CV death or HHF), change in KCCQ score at 8 months
≥20 to <45	None	EMPA-KIDNEY	Empagliflozin 10 mg	Kidney composite	Hospitalization for any cause
>45 to 90	≥22.6				
≥30	n/a	EMPA-REG OUTCOME	Empagliflozin 10 or 25 mg	3-Point MACE	n/a
≥20	n/a	EMPEROR-Reduced	Empagliflozin 10 mg	CV death or HHF	Total number of HHF events, eGFR slope
≥20	n/a	EMPEROR-Preserved	Empagliflozin 10 mg	CV death or HHF	Total number of HHF events, eGFR slope
≥25 to <75	≥3.4 to >33.9	FIDELIO-DKD	Finerenone 10-20 mg/day	Kidney composite	(3-point MACE or HHF), all-cause mortality
≥25 to <60	≥33.9 to <565				
≥25 to ≤90	≥3.4 to >33.9	FIGARO-DKD	Finerenone 10-20 mg/day	3-Point MACE or HHF	n/a
≥60	≥33.9 to <565				

*Note.* Composite secondary endpoints are grouped within square parentheses. 3-Point MACE: A composite of cardiovascular death, nonfatal myocardial infarction, and nonfatal stroke. nsMRA = nonsteroidal mineralocorticoid receptor antagonist; eGFR = estimated glomerular filtration rate; UACR = urine albumin-to-creatinine ratio; CV = cardiovascular; HHF = Hospitalization for heart failure; MACE = major adverse cardiovascular events; ESKD = end-stage kidney disease; CKD = chronic kidney disease; HF = Heart failure; KCCQ = Kansas City Cardiomyopathy Questionnaire; SGLT2 = sodium glucose cotransporter 2; n/a = not available.

a Dapagliflozin was found to be noninferior but not superior to placebo on the primary outcome in DECLARE-TIMI 58.

### Question 5. What Treatments Do We Have for Patients Who Continue to Progress Despite Treatment With Historic Standard of Care, With or Without SGLT2 Inhibitors?

Despite treatment with current standard of care, patients with CKD and T2D have high residual cardiorenal morbidity and mortality.^6,60,67-69^ Efforts have been made to assess risk based on an individual’s eGFR and albumin-to-creatinine ratio (eg, the heat map included in the KDIGO CKD guidelines).^70^ Hemodynamic factors, metabolic factors, and inflammation and fibrosis are key drivers of CKD progression in T2D. While ACEi, ARBs, and SGLT2 inhibitors target hemodynamic factors and SGLT2 inhibitors, GLP1-RAs, and other antidiabetic therapies target metabolic factors, there are no treatments available to date that are *specifically* designed to target inflammation and fibrosis.^67,71-73^ Evidence suggests that overactivation of the mineralocorticoid receptor (MR) leads to inflammation and fibrosis in the kidneys and heart, where the MR is extensively expressed, resulting in progression of CKD and CV disease.^74^

Up until recently, there had been no large cardiorenal outcome trials conducted with MRAs among patients with DKD. While their mechanism of action suggests MRAs such as eplerenone or spironolactone might be efficacious in the treatment of DKD, adverse effects including hyperkalemia and sexual side effects have limited their widespread use.^40^

Finerenone is a potent and selective nsMRA that blocks MR overactivation and inhibits expression of proinflammatory and profibrotic mediators, including those associated with CKD progression.^75-79^ Recently 2 pivotal trials examining cardiorenal endpoints and safety of finerenone in addition to RAAS inhibitors in people with DKD were published. These studies were FIDELIO-DKD and FIGARO-DKD. Their designs were complementary with similar endpoints and largely overlapping patient populations.^8,19^ In the FIDELIO-DKD trial (N = 5734), finerenone significantly reduced the risk of the primary kidney composite outcome and the key secondary CV composite outcome in patients with CKD stage 3 or 4, most with moderate or severe albuminuria.^8^ In the FIGARO-DKD trial (N = 7437), finerenone significantly reduced the primary CV composite outcome risk in patients with CKD stage 2 to 4 with moderately increased albuminuria or in those with CKD stage 1 or 2 with severely increased albuminuria.^19^ As expected due to its mechanism of action, an increase in hyperkalemia was reported with finerenone versus placebo in both trials (2.3% vs 0.9%, respectively, in FIDELIO-DKD, and 1.2% vs 0.4% in FIGARO-DKD).^8,19^

FIDELITY was a prespecified pooled analysis of the FIDELIO-DKD and FIGARO-DKD trials. Combining the 2 trials is appropriate as they share similar designs, overlapping patient populations, and endpoints (although with inverted primary and prespecified secondary endpoints). Across the patient population, the RR reductions in the composite CV and composite kidney outcomes were 14% and 23%, respectively. Hospitalization for HF was the primary driver of CV benefits with finerenone, with a RR reduction of 22% versus placebo (*P* = .0030). FIDELITY showed a 30% reduction in the risk of a sustained ≥57% decrease in eGFR and an RR reduction of 20% in ESKD with finerenone versus placebo (*P* = .0403). Hyperkalemia was, however, more frequent with finerenone versus placebo.^80^

A combination therapy is considered state of the art and is recommended in other therapeutic areas. For example, guidelines from the Canadian Cardiovascular and Canadian Heart Failure Societies recommend combination therapy with an angiotensin receptor-neprilysin inhibitor or ACEi/ARB, beta blocker, MRA, and SGLT2 inhibitor as standard of care in symptomatic patients with HFrEF.^81^

In FIDELITY, 6.7% of patients (n = 877) were receiving an SGLT2 inhibitor at baseline. Analyses suggest that the CV and kidney benefits of finerenone are at least as large in patients on SGLT2 inhibitors as in those without. An additive effect of the combination of finerenone and SGLT2 inhibitors has been suggested due to their distinct mechanisms of action; however, further studies are required to confirm this (see Question 10).^80,82^

**Practice Points:**

In patients with DKD, finerenone should be considered in combination with ACEi or ARB medications to reduce the risk of CV events and CKD progression.
In patients with DKD, finerenone may be used with or without an SGLT2 inhibitor to reduce the risk of CV events and CKD progression.

### Question 6. What Are Practical Considerations When Initiating SGLT2 Inhibitors or MRAs and Monitoring Patients With DKD?

Canadian and international guidelines recommend that patients with T2D should be screened annually for CKD through measurement of both UACR and eGFR (see Question 1), and these measures should be performed at least annually in those with confirmed CKD, depending on disease severity.^4,83^ Patients with CKD are also at increased risk of CV disease, HF, CV events, and peripheral artery disease.^84^ Clinical assessment for symptoms and signs of CV disease and HF should be conducted regularly, as well as an electrocardiogram to screen for CV disease and appropriate diagnostic testing as indicated (echocardiogram and/or B-type natriuretic peptide [BNP] or N-terminal proBNP [NT-proBNP] if appropriate); however, the role of BNP/NT-proBNP screening is uncertain especially as levels should be interpreted with caution in patients with eGFR <60 mL/min/1.73 m^2^.^84^

#### SGLT2 inhibitors

**Figure 3.**
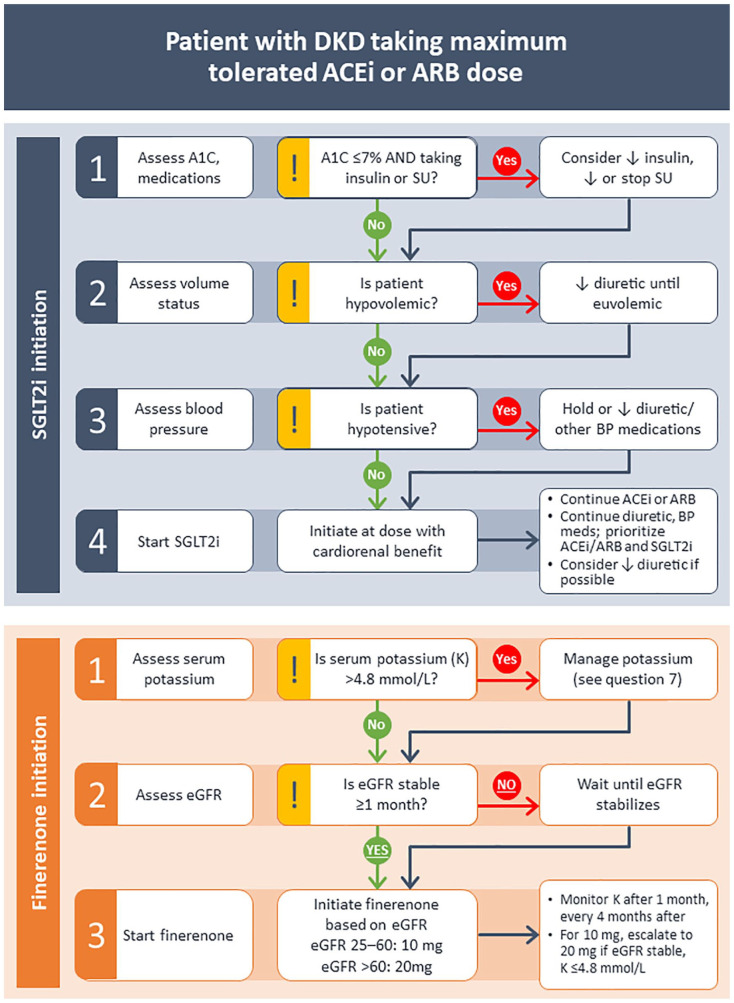
Flowcharts illustrating expert opinion for initiation of SGLT2 inhibitors and finerenone in patients with DKD. *Note.* Patients should be taking the maximum tolerated dose of an ACEi or ARB before initiation of either SGLT2i or finerenone. Note that initiation of SGLT2i and finerenone should be sequential rather than simultaneous, and no recommendation is made on the optimal order of initiation of these medications. SGLT2 = sodium glucose cotransporter 2; DKD = diabetic kidney disease; ACEi = angiotensin-converting enzyme inhibitor; ARB = angiotensin II receptor blocker; SGLT2i = sodium glucose cotransporter 2 inhibitor; T2D = type 2 diabetes; BP = blood pressure; SU = sulfonylurea.

Before initiating an SGLT2 inhibitor, the patient’s current glycemic control, BP, and volume status should be assessed. In patients with diabetes and GFR >60 mL/min/1.73 m^2^, SGLT2 inhibitors lower A1C by 0.5% to 0.7%^32^; however, the glucose-lowering effect of SGLT2 inhibition is reduced when GFR is <60 mL/min/1.73 m^2^; the risk of hypoglycemia in those on insulin or sulfonylurea therapy would therefore be expected to be less clinically significant.^85^ In patients at risk of hypoglycemia, particularly those with A1C < 7% and taking insulin and/or sulfonylurea therapy, the insulin dose should be reduced by approximately 20%, and/or sulfonylurea dose reduced or stopped at the time of SGLT2 inhibitor initiation. Consideration should also be given to adjusting other medications to correct hypovolemia before initiating an SGLT2 inhibitor, with a preference for maintaining stable RAAS inhibitor doses and reducing other antihypertensives. Adjustment of loop diuretic dosing may also be required in patients who are already euvolemic and normotensive at baseline, with the caveat that HF patients often exhibit and tolerate lower BP levels and require higher doses of loop diuretics even when starting SGLT2 inhibitors^49^ (Figure 3).

SGLT2 inhibitors for DKD should be initiated at the dose with evidence of cardiorenal benefit (ie, canagliflozin 100 mg daily, dapagliflozin 10 mg daily, empagliflozin 10 mg daily [the doses used in the prospective kidney trials; note that the dosing may have been different in earlier CV outcome trials]). Contemporary labeling specifies that eGFR should be ≥30 mL/min/1.73 m^2^ for canagliflozin initiation, ≥25 mL/min/1.73 m^2^ for dapagliflozin, and >20 mL/min/1.73 m^2^ for empagliflozin if being used for the treatment of HF.^86-88^ Importantly, initiation of an SGLT2 inhibitor induces a reversible drop in eGFR of 3 to 4 mL/min/1.73 m^2^ in 60% to 70% of individuals—an effect that is not associated with progressive long-term kidney function loss or AKI. Accordingly, there is no need to order routine blood work to check kidney function or electrolytes in most patients with eGFR >45 mL/min/1.73 m^2^, unless there is a specific clinical concern about volume depletion such as in patients with BP <120/70 mm Hg, signs/symptoms of volume depletion (eg, orthostatic symptoms), in patients taking high-dose diuretics, and perhaps in the elderly.^89^ In patients in whom this concern exists, or in those with eGFR <45 mL/min/1.73 m^2^, repeat GFR testing can be considered 2 to 4 weeks after initiation, and if there is a drop of >30%, the patient should be assessed for prerenal causes of a decline in GFR (eg, illness, diuretics, nonsteroidal anti-inflammatory drugs) that can be addressed, with repeat assessment of the GFR to ensure stabilization.

Before initiation, patients should be counseled on potential adverse effects (see Question 7 for additional details).

#### Finerenone

For patients with DKD who will also need finerenone therapy, we suggest starting this agent at least 3 to 4 weeks after the initiation of an SGLT2 inhibitor. This approach is based on the concept that as both drug classes can induce a GFR “dip” (mean change of about 3.0-3.5 mL/min/1.73 m^2^ over the first 4 months of finerenone treatment), a staggered approach should help to ensure that the new eGFR level with the SGLT2 inhibitor is stable. Importantly, in the FIDELIO-DKD trial, the rate of eGFR decline was slowed with long-term treatment, with the mean eGFR in patients treated with finerenone exceeding that of patients taking placebo from 36 months after treatment initiation.^8^ However, in the absence of acute eGFR-change data in response to co-initiation of an SGLT2 inhibitor with an MRA, we recommend to start these therapies in sequence.

Before initiating finerenone, ensure that eGFR is stable and ≥25 mL/min/1.73 m^2^ and that serum potassium is 4.8 mmol/L or less (based on the threshold used as an inclusion criterion in the clinical trials). It should be noted that patients with uncontrolled hypertension (≥160/100 mm Hg) or with New York Heart Association class II-IV HF were excluded from both FIDELIO-DKD and FIGARO-DKD.^8,19^ Nevertheless, the reduction in hospitalization for HF associated with finerenone treatment in FIGARO-DKD suggests that this treatment may be safely used in patients with HF in a manner similar to steroidal MRAs (Figure 3).

For patients with an eGFR of ≥25 to <60 mL/min/1.73 m^2^, finerenone should be initiated at 10 mg daily; with escalation to 20 mg daily in patients with stable eGFR and serum potassium ≤4.8 mmol/L. For patients with an eGFR of ≥60 mL/min/1.73 m^2^, finerenone should be initiated at 20 mg once daily. Serum potassium in clinical trials was assessed 1 month after the initiation and every 4 months thereafter. Based on the FIDELIO-DKD and FIGARO-DKD trial protocols, it is reasonable to assess serum potassium 1 month after the initiation of finerenone and then on a regular basis according to local standard of care and to temporarily hold finerenone based on the serum potassium level as per the trial protocol.^8,19^ A further discussion of hyperkalemia and its management is found in Question 7.

Interestingly, post hoc analyses from both the CREDENCE and DAPA-HF trials suggest that SGLT2 inhibitor use may reduce the risk of significant hyperkalemia, an observation that could facilitate treatment with finerenone.^53,90^ Potassium-binding agents may also help ensure patients can benefit from finerenone treatment. Before initiation, patients should be counseled on potential adverse effects. See Question 7 for additional details.

**Practice Points:**

Patients with confirmed DKD should have measures of UACR and eGFR taken every year, with more frequent measurements in patients with higher disease severity.
Initiate SGLT2 inhibitors for DKD at the dose with evidence of cardiorenal benefit (ie, canagliflozin 100 mg daily, dapagliflozin 10 mg daily, empagliflozin 10 mg daily).
In patients with DKD or HF, RAAS inhibitor and SGLT2 inhibitor initiation and maintenance should be prioritized over other medications with antihypertensive effects.
Routine eGFR and electrolyte measurements after the initiation of SGLT2 inhibitors are recommended only in cases where there is clinical concern about volume status (eg, BP <120/70 mm Hg, sign/symptoms of volume depletion, high-dose diuretics, elderly, eGFR <45 mL/min/1.73 m^2^).
When initiating finerenone, check serum potassium levels 2 to 4 weeks after the initiation and regularly thereafter.

### Question 7. What Are the Adverse Events Associated With CKD Therapies and How Are They Best Managed?

#### RAAS inhibitors

While RAAS inhibitors have several important renal and CV benefits, it is important to be aware of the potential adverse events associated with their use.

One of the most common adverse events with RAAS inhibitors is that they are known to induce or exacerbate hyperkalemia.^8,91-94^ It has also been found that having 1 episode of hyperkalemia puts patients at risk of a second episode. Predictors of hyperkalemia recurrence among patients on RAAS inhibitors include a moderate to severe first episode of hyperkalemia (potassium ≥5.6 mmol/L), low eGFR, diabetes, and the use of spironolactone.^95^

For steroidal MRAs (spironolactone and eplerenone), the incidence of hyperkalemia is nearly double that of individuals not on these agents.^96^ In contrast, a lower incidence of hyperkalemia was reported in a phase 2 study comparing the novel nsMRA finerenone to spironolactone (5.3% vs 12.7%).^96^ Nevertheless, the incidence of hyperkalemia-related discontinuation remains higher with finerenone than with placebo (2.3% vs 0.9%, respectively, in the FIDELIO-DKD trial and 1.2% vs 0.4% in the FIGARO-DKD trial).^8,19^

Hyperkalemia associated with the use of RAAS inhibitors can often be managed by measures to reduce serum potassium levels rather than immediately decreasing the dose or discontinuing these medications.^41^ Prevention and management of nonemergent hyperkalemia includes a low-potassium diet (although the utility of this intervention has been questioned)^97,98^ and discontinuation of potassium-containing supplements; the use of loop or thiazide diuretics; correction of concurrent metabolic acidosis with sodium bicarbonate; carefully dosing RAAS inhibitors and other medications that may elevate potassium; and the use of potassium binders.^99,100^

A growing body of evidence supports using the newer potassium binders, sodium zirconium cyclosilicate (SZC) and patiromer, to enable the use of RAAS inhibitors.^101-104^ For example, the HARMONIZE trial demonstrated that SZC given to outpatients with hyperkalemia successfully lowered and maintained normokalemic potassium levels for 28 days in a population with 70% RAAS inhibitor use at baseline.^105^ The OPAL-HK (4-week follow-up) and AMETHYST-DN trials (52-week follow-up) showed that patiromer enabled maintenance of normal serum potassium levels in a population with eGFR <60 mL/min/1.73 m^2^ and 100% baseline RAAS inhibitor use.^103,106^ In the PEARL-HF trial, there was a significantly lower incidence of hyperkalemia among patients with HF randomized to patiromer (7.3% vs 24.5% patients randomized to placebo), which enabled greater use of spironolactone 50 mg/day (91% vs 74%).^102^ Similarly, in the AMBER trial, among patients with CKD and resistant hypertension, a greater number of patients treated with patiromer were able to continue spironolactone with less hyperkalemia than those on placebo.^104^ Overall, these studies show that the use of the novel potassium binders can mitigate the risk of hyperkalemia and, thus, allow patients to maintain the benefits of being on RAAS inhibitors.

Gynecomastia is another common side effect of spironolactone, with the overall incidence estimated at 11%.^107^ In the RALES trial, gynecomastia was more frequently reported with spironolactone versus placebo (10% vs 1%).^108^ Compared with the use of ACEis and ARBs, spironolactone was found to increase the risk of gynecomastia approximately 5-fold.^109^ In comparison, studies of eplerenone and finerenone have reported no increased incidence of gynecomastia.^8,19,110^

#### SGLT2 inhibitors

SGLT2 inhibitors are associated with a wide variety of metabolic and cardiorenal benefits and are included in numerous clinical practice guidelines.^32,111^ Despite being generally well tolerated, adverse events associated with SGLT2 inhibitors can include genital mycotic infections (GMIs), urinary tract infections (UTIs), possible risk of amputation, and diabetic ketoacidosis (DKA).^112^

Patients on SGLT2 inhibitors have been reported to have a 3- to 4-fold increased risk of GMIs. However, these infections are typically not severe and only rarely require treatment discontinuation.^113^ Patients should, however, be counseled on the increased risk of GMIs associated with SGLT2 inhibitors; prescribers may consider providing additional counseling on genital hygiene as this may reduce incidence and severity of GMI.^114,115^ If a patient experiences a GMI, the recommended treatment is a single dose of oral fluconazole 150 mg.^116^

T2D is associated with an increased risk of UTI. The risk of UTI is also 2 to 3 times higher in women than in men and more common in people older than 60 years. While 1 SGLT2 inhibitor trial found an increase in UTIs, meta-analyses suggest that there is no statistically significant increase in severe UTIs. Patients with T2D should, however, be aware of the potential risk of UTIs and seek medical advice should symptoms occur.^117^

Patients enrolled in the CANVAS and CANVAS-R trials experienced a 1.97-fold increase in amputation risk associated with canagliflozin use, with the highest absolute risk of amputation among patients with a history of amputation or peripheral vascular disease.^64^ As a result, standardized foot care was included in the CREDENCE trial protocols, and medication was temporarily interrupted for patients with any active condition that might lead to amputation. No significant differences in amputation rates were seen.^6^ A meta-analysis of the effects of SGLT2 inhibitors on kidney outcomes across 13 large placebo-controlled trials found no association between SGLT2 inhibitors and lower-limb amputation when CANVAS/CANVAS-R was excluded (RR 1.06, 95% CI 0.93-1.21; heterogeneity for CANVAS vs other 12 trials, *P* = .0007), and no association was found in an analysis specific to patients with CKD and diabetes (RR 1.05, 95% CI 0.84-1.32).^48^ Nonetheless, good foot care is recommended in all patients with T2D.^32^

DKA is rare in patients taking SGLT2 inhibitors.^66^ A systematic review and meta-analysis of RCTs found that SGLT2 inhibitors have been associated with cases of DKA in patients with diabetes, but not in patients without diabetes. Analysis of 13 placebo-controlled trials with median follow-ups ranging from 0.8 to 4.2 years (n = 74 804 patients with diabetes) found that in the reported 167 DKA events, SGLT2 inhibitors were associated with an increased risk of DKA versus control (reported rate in placebo groups of 0.2 events per 1000 patient years; RR for SGLT2 inhibitors of 2.12, 95% CI 1.49-3.04).^48^ Subgroup analyses showed a larger relative effect among patients aged ≥60 years and those with longer use of SGLT2 inhibitors (>52 weeks).^118^

SGLT2-inhibitor-associated DKA is more likely in patients with insulin-deficient diabetes, including those with T2D, and may present with euglycemic DKA due to the glucosuric effect of SGLT2 inhibition. DKA is typically precipitated by insulin omission or dose reduction, severe acute illness, dehydration, surgery, low-carbohydrate diets, or excessive alcohol intake. DKA associated with SGLT2 inhibitor use may be avoided by withholding SGLT2 inhibitors when precipitants occur or prior to a planned surgery, avoiding insulin omission or inappropriate insulin dose reduction, and by following sick day protocols.^119^

A preplanned systematic review of population-based studies investigating SGLT2 inhibitor effectiveness and safety in T2D found that in 37 studies (total N = 1 300 184; total follow-up 910 577 person-years), canagliflozin, dapagliflozin, and empagliflozin were not significantly associated with an increased risk of AKI (point estimate range hazard ratio [PER HR] 0.40-0.96), fractures (PER HR 0.87-1.11), hypoglycemia (PER HR 0.76-2.49), or UTI (PER HR 0.72-0.98).^120^ Additionally, a meta-analysis of the effects of SGLT2 inhibitors on kidney outcomes across 13 large placebo-controlled trials found that treatment with SGLT2 inhibitors was associated with a reduced incidence of AKI among patients with diabetes (RR 0.79, 95% CI 0.72-0.88).^48^

#### Sick day medications

Medications commonly used in the management of DKD can reduce kidney function in patients experiencing an intercurrent illness. These medications should be temporarily discontinued, especially in patients with reduced oral intake or excessive losses due to vomiting or diarrhea, leading to hypovolemia. Patients should be counseled on sick day medications and when they should be avoided. The Diabetes Canada Sick Day Medication List includes sulfonylureas, ACEi, diuretics and direct renin inhibitors, metformin, ARBs, nonsteroidal anti-inflammatory drugs, and SGLT2 inhibitors (SADMANS).^32^

**Practice Points:**

RAAS inhibitors should be titrated to the highest approved, tolerated dose. Lowering of the dose or discontinuing RAAS inhibitors to lower potassium levels or prevent additional episodes of clinically significant hyperkalemia should only be undertaken after attempting measures to maintain the evidence-based dose of RAAS inhibitor.
Patients with diabetes who are prescribed SGLT2 inhibitors should be educated about the signs and symptoms of GMI and DKA.
Patients should be counseled on sick day medications (SADMANS) and when they should be avoided.

### Question 8. How Do We Identify Appropriate Patients and Incorporate Newer Treatments Into the Management of DKD?

Evaluation of kidney function or markers of kidney damage in patients with confirmed T2D should be done at the time of diagnosis and at least annually thereafter by measurement of urinary albumin excretion (best done by a UACR) and serum creatinine with an eGFR (see Question 1).^4^ Upon diagnosis of DKD, patients should be offered a comprehensive strategy to reduce the risk of kidney disease progression and prevent or reduce CV and vascular disease.^41^ Treatment decisions should be individualized based on patients’ cardiorenal risk, preferences, access and cost, and degree of glucose lowering needed.^32^

The cardiorenal benefits of SGLT2 inhibitors are now well established. SGLT2 inhibitors reduce the risk of kidney disease progression, hospitalization for HF, and CV events.^32^ SGLT2 inhibitors are recommended for most patients with T2D and CKD with few exceptions. SGLT2 inhibitors are not indicated in patients with type 1 diabetes; prior experience of DKA can also be considered a relative contraindication. A risk-benefit assessment should be made in patients with frequent UTIs or fungal genital infections, indwelling urinary catheters, active foot infections, or at high risk of volume depletion.^41^ New data are being explored in the use of SGLT2 inhibitors among patients with T2D who have undergone kidney transplantation. SGLT2 inhibitors can be started in patients with eGFRs of at least 30 mL/min/m^2^ for canagliflozin and empagliflozin and 25 mL/min/m^2^ for dapagliflozin. SGLT2 inhibitors can be continued until an eGFR of 15 mL/min/m^2^ or the start of dialysis (see Question 3, 4, and 6).^6,7,121^ Empagliflozin is indicated for treatment of HF with SGLT2 inhibitor initiation at eGFRs ≥20 mL/min/m^2^.^57,58^

GLP1-RAs also have an important place in the management of DKD. The GLP1-RAs dulaglutide, liraglutide, and semaglutide have been associated with a reduction in 3-point MACE, and these benefits appear to be maintained in patients with kidney impairment. GLP1-RA use reduces albuminuria; however, the effect on “hard” kidney outcomes (eg, doubling of serum creatinine or significant eGFR decrease, progression to ESKD, or kidney death) requires further validation (see Question 10). GLP1-RAs have other attributes: They do not require dose adjustments in patients with low eGFRs, and they retain the ability to reduce A1C levels in patients with kidney impairment. Additionally and importantly, GLP1-RA use results in significant weight loss (>5% of initial weight) in the majority of patients.^41,122^ Obesity is quickly becoming a contributor to worsened kidney function, exacerbating preexisting comorbidities such as hypertension and diabetes. Obesity is associated with delays in patients getting on a list for kidney transplantation and in receiving a transplant once on a list, as there is a higher risk of graft dysfunction.^41^

CKD progression in T2D is driven by the combined effects of metabolic, hemodynamic, and inflammatory and fibrotic factors.^67,71,72^ SGLT2 inhibitors act on metabolic, hemodynamic, and likely inflammatory pathways, and GLP-1RAs act on the metabolic pathway; however, finerenone is believed to act *specifically* on inflammation and fibrosis by blocking overactivation of MR.^68,111,123^ Clinical data suggest that finerenone may offer cardiorenal benefits in patients with T2D and eGFR ≥25 mL/min/1.73 m^2^ with proteinuria (see Question 5).^8,19^

**Practice Points:**

Treatment decisions should be individualized based on risks and benefits, patient needs and preferences, access and cost, and the degree of glucose lowering needed.
For most patients with DKD who need additional glycemic control or who are at high CV risk, a GLP1-RA should be considered.

### Question 9. What Is the Place of Primary Care in Identification and Treatment of DKD?

Primary-care practitioners bear most of the responsibility both for diagnosing diabetes and for providing care (including the management of DKD) to those who are diagnosed; an estimated 80% of medical care for Canadians with diabetes is the responsibility of primary care.^124^

This brief section will provide an overview of the key responsibilities of primary-care practitioners in diabetes screening in general, screening for DKD in particular, and providing treatment regimens to optimize cardiorenal protection, with particular focus on the newer additions to the risk-reduction armamentarium, SGLT2 inhibitors and finerenone.

#### Screening for diabetes and DKD

Current Diabetes Canada guidelines recommend that all individuals aged 40 years or older (as well as individuals identified as being at high risk of diabetes on a risk calculator) should be screened for T2D using fasting plasma glucose and/or A1C every 3 years.^125^

Diabetes Canada also provides clear recommendations for screening for DKD, with both an eGFR and a measurement of albuminuria (see Question 1, Figure 1). The use of both these screening tools in primary care in Canada has been suboptimal (see Question 1), with the use of albuminuria screening being substantially lower than that of eGFR. Both measurements are required not only for the identification of DKD but also for classification, assessment of prognosis (ie, risk of cardiorenal morbidity and mortality), and tracking of disease progression.^83,125^

There are several potential barriers to the uptake of UACR testing in primary care. A cross-sectional survey of 165 US primary-care practitioners conducted between April and June 2013 provided some insight into these barriers.^126^ Asked specifically about what would constitute a barrier to using urinary albumin testing in patients with an eGFR <60 mL/min/1.73 m^2^, 24% of respondents selected “no impact on management.” Other selected responses were “limited time/more urgent patient issues” (20% of respondents), “not recommended by guidelines” (11%), “cost” (9%), and “poor patient adherence” (5%).^126^

One approach that may be helpful to increase the uptake of UACR testing in primary care is to harness the potential of electronic medical records (EMRs).^127^ Some Canadian provinces provide financial incentive to physicians to practice evidence-based chronic disease management of diabetes, and many EMRs include a template that can be completed to help facilitate this. Expanding compensation and providing more robust support through EMR systems have the potential to improve screening.

#### Prescribing renoprotective medications

Primary-care providers are uniquely positioned to help ensure optimal use of risk-reducing interventions for their patients with diabetes. With respect to DKD, until recently, guideline-recommended risk-reducing approaches were optimal management of blood glucose and BP and use of RAAS inhibitors (see Question 2).^4^ Canadian research has suggested there is still room for improvement in maximizing the uptake of these well-known interventions. An analysis of routine laboratory and administrative data in Alberta from 2015 to 2017 by the Kidney Health Strategic Clinical Network showed that among patients with diabetes and CKD stage 3 or 4 (as measured by eGFR), 77.7% were taking an ACEi or ARB.^27^ While this does illustrate a treatment gap, practitioners may take some comfort in knowing that this gap is even more striking in other developed countries. Analysis of the CURE-CKD registry in the United States, for example, showed that only about 20% of CKD patients in the registry were taking an ACEi or ARB, and the rates were even lower among the subgroup of CKD patients with either diabetes or prediabetes.^128^

For the kidney-protective pharmacotherapies discussed extensively in this article (SGLT2 inhibitors and nsMRAs), primary-care physicians will continue to have a critical role to play in prescribing and monitoring their use. SGLT2 inhibitors are agents that most primary-care practitioners are already familiar with, having been available for use in Canada as antihyperglycemic agents since 2014.^129^ Evolving evidence has established SGLT2 inhibitors among the core recommended therapies for many indications beyond A1C lowering, including the reduction of cardiorenal outcomes among several different patient groups both with and without T2D.^32,41,81^ SGLT2 inhibitors are simple medications to use in primary care, with minimal pretesting or laboratory follow-up required, and primary-care practitioners should be involved in ensuring that all appropriate patients with DKD (see Questions 3 and 8) are receiving these agents.

Regarding nsMRAs, although most primary-care practitioners are familiar with spironolactone, finerenone is new to the treatment landscape in Canada, approved in October 2022. Landmark trials indicate that finerenone also reduces cardiorenal outcomes in patients with DKD.^8,19^ The use of finerenone, for appropriate patients (see Questions 5 and 8) will likely follow a similar pathway as was seen with other innovative therapies, with initial uptake led by specialists as any uncertainties and finer points of use are elucidated (including appropriate potassium management), before more widespread use in primary care. Indeed, the lessons learned and pathways developed for SGLT2 inhibitor uptake may provide a reasonable framework for the incorporation of finerenone into the overall treatment paradigm for DKD.

While early identification of DKD is important (see Question 1), it is critical to note that the interventions for cardiorenal protection identified in this document, including SGLT2 inhibitors and finerenone, can provide benefits for most patients with DKD, regardless of stage.

**Practice Points:**

Primary-care practitioners should screen adult patients for diabetes, following the current Diabetes Canada guidelines.
Primary-care practitioners should follow screening guidelines for DKD including measurement of both eGFR and albuminuria as discussed in Question 1.
Primary-care practitioners should prescribe SGLT2is for appropriate patients with DKD, following the evidence and practice points in Questions 3 and 8.
For patients who may benefit from finerenone therapy (see practice points in Questions 5 and 8), primary-care physicians should consider consulting with a specialist to determine individual patient suitability.

### Question 10. What Are the Ongoing Research Initiatives and Gaps in DKD Moving Forward?

One important limitation of consensus statements and guidelines is the constant evolution of the relevant body of evidence, making it difficult to remain current and relevant. In this section, we aim to summarize a selection of ongoing studies that are likely to apply to the content of this consensus statement. Updates to this document and additional recommendations, if required, are planned to be housed on https://ukidney.com, a Canadian website with educational content in nephrology.

As noted above, primary kidney outcome data are currently lacking for the GLP1-RA class. While a reduction in albuminuria and new-onset macroalbuminuria has been associated with this class in meta-analyses of data reported from CVOTs, reductions have not been observed in “hard” kidney outcomes, such as doubling of serum creatinine, incident ESKD, or death from renal causes.^60^ The FLOW trial, set to report in 2024, will test whether treatment with semaglutide reduces a primary composite kidney outcome consisting of persistent eGFR decline of ≥50% from trial start, incident ESKD, or death from kidney or CV causes.^130^

Additional data are also needed on SGLT2 inhibitor combination therapy. A pooled subanalysis of the FIDELIO-DKD and FIGARO-DKD trials suggests that the combination of SGLT2 inhibitors and finerenone may provide an additive reduction in kidney outcomes, but the potential superiority of the combination therapy over either medication on its own is yet to be proven.^82^ Similarly, meta-analysis suggests that effects of GLP1-RA and SGLT2 inhibitors on A1C, body weight, and BP are additive, and there is some indication that CV benefits are additive.^131^ The largest cohort of patients taking an SGLT2 inhibitor and GLP1-RA in a CVOT to date is from the AMPLITUDE-O trial, which showed that the reduction in 3-point MACE in patients taking efpeglenatide was consistent regardless of whether patients were taking an SGLT2 inhibitor at baseline.^132^ A systematic, prospective evaluation of CV or renal benefits associated with the combination of GLP1-RA and SGLT2 inhibitor medication compared to each medication on its own might be helpful in defining any potential benefit of using both medications in high-risk patients, as has been endorsed for high-risk patients in some guidelines outside of Canada.^111^

The kidney benefits of SGLT2 inhibitors and finerenone are well proven in DKD. The demonstrated slowing of eGFR decline and reduction in incident ESKD are almost certainly indicative of an important pharmacoeconomic benefit. A formal analysis of return on investment in prescribing these medications would help bolster the case for formulary inclusion and improve access so that more patients may benefit from their effects.

## Limitations

This review was developed through a process of consultation, discussion, and debate among a multidisciplinary panel of experts (9 nephrologists, an endocrinologist, and a primary-care practitioner). No formal guideline process such as Grading of Recommendations, Assessment, Development and Evaluations (GRADE) was used.^133^ As a result, the practice points are not graded and are not intended to be viewed as having the weight of a clinical practice guideline or formal consensus statement. However, most practice points are well aligned with clinical practice guidelines such as those published by the ADA, Canadian Diabetes Association, and KDIGO.^4,21,41^

## Conclusions

See Table 3 below.

**Table 3.** Summary of Practice Points.

Patients with diabetes should be routinely screened for DKD with assessments of urinary albumin and kidney function, following current Diabetes Canada guidelines.
Patients with DKD should be treated to achieve targets for A1C, following current guidelines from Diabetes Canada.
Patients with DKD should be treated to achieve a target BP following current guidelines from Diabetes Canada and Hypertension Canada.
Patients with DKD should be treated with a RAAS inhibitor (either an ARB or ACEi).
For patients with DKD and eGFR >20 mL/min/1.73 m^2^, treatment with an SGLT2 inhibitor can be initiated as part of the standard of care along with ACEi/ARB use, BP control, and A1C control.
For patients who progress to ESKD requiring dialysis, SGLT2 inhibitors should be discontinued.
In patients with a history of heart failure, SGLT2 inhibitors should be initiated to reduce the risk of CV death or hospitalization for heart failure.
In high-risk patients with T2D (ie, those with ASCVD and/or CKD), SGLT2 inhibitors should be initiated to reduce the risk of cardiovascular events.
In patients with DKD, finerenone should be considered in combination with ACEi or ARB medications to reduce the risk of CV events and CKD progression.
In patients with DKD, finerenone may be used with or without an SGLT2 inhibitor to reduce the risk of CV events and CKD progression.
Patients with confirmed DKD should have measures of UACR and eGFR taken every year, with more frequent measurements in patients with higher disease severity.
Initiate SGLT2 inhibitors for DKD at the dose with evidence of cardiorenal benefit (ie, canagliflozin 100 mg daily, dapagliflozin 10 mg daily, empagliflozin 10 mg daily).
In patients with DKD or heart failure, RAAS inhibitor and SGLT2 inhibitor initiation and maintenance should be prioritized over other medications with antihypertensive effects.
Routine eGFR and electrolyte measurements after the initiation of SGLT2 inhibitors are recommended only in cases where there is clinical concern about volume status (eg, BP <120/70 mm Hg, sign/symptoms of volume depletion, high-dose diuretics, elderly, eGFR <45 mL/min/1.73 m^2^).
When initiating finerenone, check serum potassium levels 2 to 4 weeks after initiation and regularly thereafter.
RAAS inhibitors should be titrated to the highest approved, tolerated dose. Lowering of the dose or discontinuing RAAS inhibitors to lower potassium levels or prevent additional episodes of clinically significant hyperkalemia should only be undertaken after attempting measures to maintain the evidence-based dose of RAAS inhibitor.
Patients with diabetes prescribed SGLT2 inhibitors should be educated about the signs and symptoms of GMI and DKA.
Patients should be counseled on sick day medications (SADMANS) and when they should be avoided.
Treatment decisions should be individualized based on risks and benefits, patient needs and preferences, access and cost, and the degree of glucose lowering needed.
For most patients with DKD who need additional glycemic control or who are at high CV risk, a GLP1-RA should be considered.
Primary-care practitioners should screen adult patients for diabetes, following the current Diabetes Canada guidelines.
Primary-care practitioners should follow screening guidelines for DKD including measurement of both eGFR and albuminuria as discussed in Question 1.
Primary-care practitioners should prescribe SGLT2is for appropriate patients with DKD, following the evidence and practice points in Questions 3 and 8.
For patients who may benefit from finerenone therapy (see practice points in Questions 5 and 8), primary-care physicians should consider consulting with a specialist to determine individual patient suitability.

*Note.* DKD = diabetic kidney disease; A1C = glycated hemoglobin; BP = blood pressure; RAAS = renin-angiotensin-aldosterone system; ARB = angiotensin II receptor blocker; ACEi = angiotensin-converting enzyme inhibitor; eGFR = estimated glomerular filtration rate; SGLT2i = sodium glucose cotransporter 2 inhibitor; ESKD = end-stage kidney disease; CV = cardiovascular; T2D = type 2 diabetes; CKD = chronic kidney disease; UACR = urine albumin-to-creatinine ratio; GMI = genital mycotic infection; DKA = diabetic ketoacidosis; GLP1-RA = glucagon-like peptide-1 receptor agonist; ASCVD = atherosclerotic cardiovascular disease.
